# Effectiveness and intention to use a COVID-19 self-management app for epidemiological investigation: a web-based survey study

**DOI:** 10.3389/fpubh.2024.1343734

**Published:** 2024-03-27

**Authors:** Sihyun Song, Jihwan Park, Mi Jung Rho

**Affiliations:** ^1^Department of Healthcare Service Management, Graduate School of Health and Welfare, Dankook University, Cheonan-si, Chungcheongnam-do, Republic of Korea; ^2^College of Liberal Arts, Dankook University, Cheonan-si, Chungcheongnam-do, Republic of Korea; ^3^College of Health Science, Dankook University, Cheonan-si, Chungcheongnam-do, Republic of Korea

**Keywords:** COVID-19, users’ assessment, application, epidemiological investigation, intention to use

## Abstract

**Introduction:**

Numerous COVID-19-related apps were widely used during the COVID-19 pandemic. Among them, those supporting epidemiological investigations were particularly useful. This study explored the effectiveness of apps that support epidemiological investigations, factors influencing users’ intention to use them, and ways to encourage their use.

**Methods:**

We developed and evaluated the KODARI app to demonstrate its importance in epidemiological investigations. After adapting a questionnaire based on an existing evaluation framework for COVID-19–related apps, we collected data from 276 participants through an online survey conducted between April 28 and May 25, 2023. We conducted two independent sample *t*-tests to determine the differences between each variable according to demographic characteristics and a multiple regression analysis to identify factors affecting intention to use.

**Results:**

Users were generally satisfied with the KODARI. We observed differences in sex, age, marital status, occupational characteristics, and experience with epidemiological investigation. Females rated the app’s information accuracy higher than males. Males had a higher intention to use than females. Participants aged under 35 years rated information accuracy and transparency highly, whereas single participants rated information accuracy higher than married participants. Occupational groups with frequent interactions with others evaluated their self-determination regarding the application. The app’s self-determination was highly valued among participants with experience in epidemiological investigations. By investigating the factors affecting the intention to use the app, we confirmed that effectiveness, self-determination, and usability significantly affected the intention to use.

**Discussion:**

This study demonstrated the effectiveness of app supporting epidemiological investigations, identified meaningful factors that influence intention to use, and confirmed the applicability of our new framework by considering the specificity of infectious disease situations such as COVID-19. This study provides a new basis for future epidemiological studies.

## 1 Introduction

The first cases of SARS–CoV-2 virus emerged in December 2019, and the global outbreak of coronavirus disease 2019 (COVID-19) was declared a pandemic by the World Health Organization (WHO) in March 2020 (1). According to a WHO report, the number of confirmed COVID-19 cases worldwide was approximately 770 million [as of August 30, 2023, Central European Summer Time (CEST)] (2). In the Republic of Korea (South Korea), the cumulative number of confirmed COVID-19 cases was approximately 34 million, and the cumulative deaths were approximately 35,000 as of August 31, 2023 (3). In South Korea, COVID-19 was legally designated and managed as a class 1 infectious disease from January 20, 2020, to April 24, 2022 (4). Epidemiological investigation is essential in case of a class 1 infectious disease. However, epidemiological investigations were limited when many COVID-19 confirmed cases occurred. The development and implementation of mobile health (mHealth) technology for epidemiological investigations is necessary.

Several mobile applications (apps) have been developed to reduce the risk of COVID-19. In addition, numerous studies have evaluated COVID-19-related apps that provide information such as news, tracking and mapping of confirmed cases, self-isolation support, and online counseling with health authorities (5, 6), and have analyzed their advantages and limitations (7). However, this technology has limitations in terms of voluntary use, and is difficult to use during epidemiological investigations.

Most epidemiological investigation apps are related to contact-tracing applications. Studies have been conducted on epidemiological investigation apps, such as COVID-19 epidemiology and tracking apps (8), Bluetooth-based user tracking, and infection risk apps (9). However, studies on the factors influencing the effectiveness and adoption of epidemiological investigations are rare.

Despite the emergence and evolution of COVID-19-related mobile apps, there is a risk of disease X threatening humanity. Disease X is a potential disease that has not been discovered (10). Additionally, WHO emphasizes international cooperation against new hypothetical infectious diseases that may emerge in the future and cause outbreaks, epidemics, or pandemics. Worldwide, preparations are underway to treat Disease X. Therefore, countermeasures against future outbreaks of Disease X, such as epidemiological investigations and support systems, are required.

We recognized the importance of an epidemiological investigation support system in the event of a new infectious disease such as Disease X. We confirmed the need to develop an epidemiological investigation app in our previous study (11) and an infectious disease self-management app for epidemiological investigations (i.e., the KODARI app) (12).

In this study, we evaluated whether the KODARI app works properly for epidemiological investigations and the prevention and treatment of infectious diseases. Additionally, we evaluated the factors that could lead to continued intention to use the app.

## 2 Materials and methods

### 2.1 COVID-19 self-management application for epidemiological investigations

We developed two COVID-19 self-management apps for epidemiological investigations: the KODARI and MEDARI apps. The KODARI app is the Korean version of an application that can be used in South Korea. The MEDARI app can be used in English-speaking countries. The main functions of the two apps are the same, with the only difference being the GPS-based location-storage map method.

This study focused on users of the KODARI app. The goal of the app is to support epidemiological investigations to minimize the spread of infectious diseases and to support individual health management. The KODARI and MEDARI apps were developed for the aforementioned purpose and designed to overcome infectious diseases through their universal use. The app database was designed for research purposes and developed based on cloud services. These two apps were not registered in the App Stores. This study was conducted with actual users prior to the official launch of the app. The total number of users was 489, among them 276 participated in this study.

**Figure 1:**
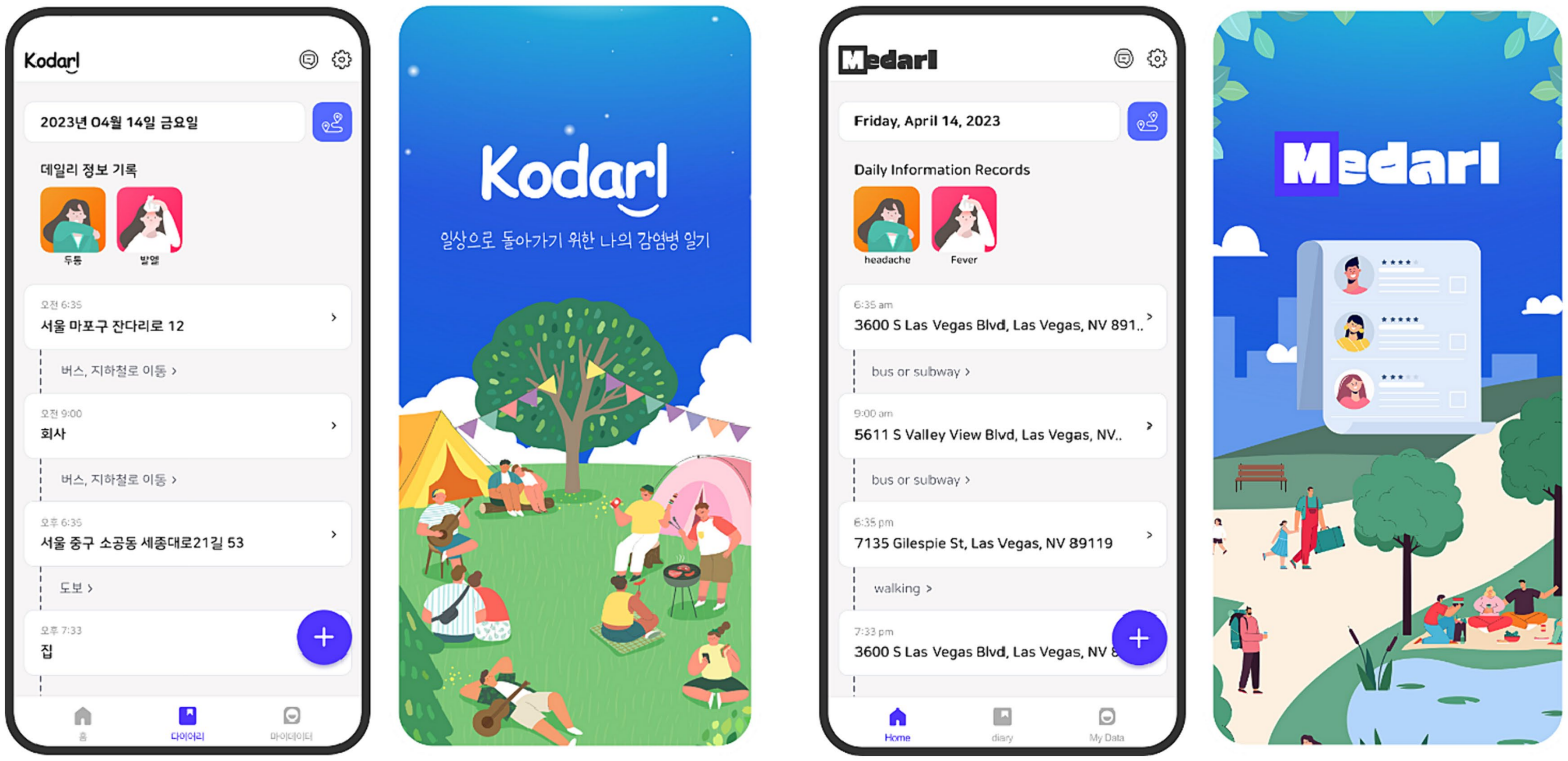
The KODARI app and the MEDARI app.

The crucial information to be collected during epidemiological investigations was extracted from the Epidemiological Investigation Form for COVID-19 (version 6) (for public health) (13). We developed the KODARI and MEDARI apps using crucial information. The main functions include daily movement routes (information on means of transportation, location, companions, etc.), daily symptom records, export of movement routes to epidemiological investigators, infectious disease management records (vaccines, test results, and exception certificates), overseas entry and exit records, and health-related information, such as underlying diseases. The detailed functions and interface of the KODARI app can be found on the KODARI (12) and MEDARI (14) websites (see Figure 1).

### 2.2 Participants

Online surveys were conducted among users of the KODARI app a COVID-19 self-management application, for epidemiological investigations. This app was released on November 1, 2022. This study targeted individuals who had used the KODARI app for more than a week (from November 2022 to the time of the survey). None of the respondents withdrew from the study.

Participants provided voluntary online consent to participate in the study prior to the online survey. The participants were compensated. The participants were given 450 points according to the survey company’s regulations on Data Spring.

### 2.3 User assessment questionnaire

This study adapted a previous assessment framework to evaluate the KODARI app (15). The seven main domains of the existing assessment framework from epidemiological and legal perspectives (15) are *purpose, usability, information accuracy, organizational attributes and reputation, transparency, privacy, and user control and self-determination*. Each domain has 40 criteria, ranging from a minimum of four to a maximum of seven items.

However, because the existing framework is a checklist for *app developers*, there are limitations to using it to conduct surveys of *app users*. Therefore, we deleted and supplemented the criteria to adapt the framework for use in our survey of KODARI app users, including changing some phrases when modifying the questionnaire for South Korean users and adjusting the framework to facilitate user (rather than developer) responses. In addition to the main elements of the existing evaluation framework, some items specific to this study were added, such as the *intention to use* and *effectiveness*. We added a *demographic characteristic* item that included basic user information, such as sex, age, and location, as well as information related to COVID-19, such as the user experience of COVID-19 testing and vaccination. The questionnaire items are included in Appendix Table A1.

The process of developing the questionnaire for this study for KODARI app users was as follows: First, a questionnaire based on an existing framework for application developers was modified for application users (15). Items regarding variables that were irrelevant to app users were deleted or supplemented. For example, an *item* referred to as *criteria* and *variable* was referred to as *domain* in a previous study. Consequently, 50 items were derived from the existing framework for seven domains: six items for purpose, five for usability, four for information accuracy, five for organizational attributes and reputation, six for transparency, seven for privacy, and seven for user control and self-determination. In addition, we added two variables based on relevant literature: intention to use and effectiveness. The total number of items, including the demographic characteristics, was 63.

The variables not bound by the factor analysis included purpose, organizational attributes, reputation, and privacy. Excluding these three variables, for the remaining six variables, our analysis used *usability* for two of the five items, *information accuracy* for two of the four items, *transparency* for two of the six items, *self-determination* for three of the seven items, *effectiveness* for three of the six items, and *intention to use* for three of the four items. To evaluate the application of the KODARI, four of the six variables were based on the existing research framework (15): usability, information accuracy, transparency, and self-determination. These six variables are defined as follows:

- *Usability* is the degree to which users perceive an app as intuitive and easy to use (15). These include application interfaces, intuitive functions, notifications, and usability for infectious disease management.
- *Information accuracy* is the degree to which users believe that data collected or provided by an app are accurate, valid, and reliable. We investigated the accuracy of the data generated, collected, and provided by the KODARI app, and whether regular updates were provided.
- *Transparency* is the degree to which users perceive that the data collected by an app are used for the purpose of the app. We investigated whether the KODARI app required minimal functionality and access and whether the app users were informed of data collection items or potential risks.
- *Self-determination* is the degree to which users perceive that they can access and control their data using an application. We investigated whether users of the KODARI app perceived that they could store, process, select, retrieve, decide, and share data on their own.
- *Intention to use* is the degree to which a user has a behavioral intention to use the app (16, 17), that is, whether users are willing to use an app. We investigated whether KODARI users were able to manage movement and health information through the app, and their likelihood of continuously using the app and recommending it to acquaintances.
- *Effectiveness* is the degree to which users perceive the app as effective in the event of an infectious disease outbreak, such as COVID-19 (18). We investigated whether KODARI users considered the app helpful in the event of a new infectious disease and reduced the work of epidemiologists and the extent to which it would do so.

### 2.4 Online survey

We conducted an online survey of KODARI users during the study. Among the existing app users, the final sample included 284 participants who volunteered to participate in the study. The survey was conducted between April 28 and May 25, 2023. Eight KODARI users who did not participate in the survey and one who responded to the survey without using the KODARI app were excluded from analysis. In total 276 valid questionnaires were completed. The survey was provided through an online link and was conducted after obtaining consent from all participants before the survey began.

### 2.5 Statistical analysis

To verify the reliability and validity of the questionnaire used in this study, we conducted factor and frequency analyses of six factors (intention to use, effectiveness, self-determination, information accuracy, transparency, and usability) to evaluate the overall KODARI app. We conducted two independent-sample *t*-tests to compare the demographic characteristics of the groups. The target variables were intention to use, effectiveness, self-determination, information accuracy, transparency, and usability, according to demographic characteristics. Finally, we investigated the factors affecting the intention to use the KODARI app using a multiple regression analysis. Multiple regression analysis was conducted using the *enter* method, in which the five independent variables (effectiveness, self-determination, information accuracy, transparency, and usability) were simultaneously input, and data were analyzed using the IBM® SPSS® Statistics 27.0 software package.

### 2.6 Ethics statement

The study procedures were performed in accordance with the Declaration of Helsinki and approved by the Institutional Review Board of D University (IRB number: DKU 2023–02–011-004). Participants’ data were de-identified.

## 3 Results

### 3.1 Participant demographics

Table 1 shows the demographic characteristics of the participants in this study evaluating the effectiveness of the KODARI application. The total number of participants is 276.

**Table 1:** Participants’ characteristics.

Variables		Frequency (%)
Sex	Male	137 (49.6)
	Female	139 (50.4)
Age	Under 19 years	2 (0.7)
	20–29 years	91 (33.0)
	30–39 years	81 (29.3)
	40–49 years	94 (34.1)
	Over 50 years	8 (2.9)
Marital status	Married (including divorced, widowed, or separated.)	134 (48.6)
	Single	142 (51.4)
Education	High school graduation or lower	28 (10.1)
	College student	47 (17.0)
	College graduation or higher	201 (72.8)
Occupation	Professional/managerial/office job	141 (51.1)
	Self-employed/freelancer	18 (6.5)
	Service/sales/production	30 (10.9)
	Homemaker/student	71 (25.7)
	Unemployed	12 (4.3)
	Other	4 (1.4)
Occupational characteristics	Occupation involving frequent interactions with others	142 (51.4)
	Occupation involving relatively fewer interactions with others	134 (48.6)
Location	Capital area	163 (59.1)
	Non-capital area	113 (40.9)
Experience of COVID-19 testing	Yes	259 (93.8)
	No	17 (6.2)
COVID-19 confirmed experience	Yes	187 (67.8)
	No	89 (32.2)
Experience with epidemiological investigation	Yes	105 (38.0)
	No	171 (62.0)
Form of epidemiological investigation	Epidemiological investigation	30 (10.9)
	Simple investigation	75 (27.2)
	Epidemiological investigation not conducted	171 (62.0)
COVID-19 reinfection	Yes	19 (6.9)
	No	257 (93.1)
Underlying diseases	Yes	25 (9.1)
	None	251 (90.9)
Vaccination	Unvaccinated	10 (3.6)
	1^st^ dose	2 (0.7)
	2^nd^ dose	86 (31.2)
	3^rd^ dose	166 (60.1)
	4^th^ dose or more	12 (4.3)
Total		276 (100.0)

Capital area: Seoul, Gyeonggi-do, Incheon Non-capital area: Busan, Daegu, Gwangju, Daejeon, Ulsan, Sejong, Gangwon-do, Chungcheongbuk-do, Chungcheongnam-do, Jeollabuk-do, Jeollanam-do, Gyeongsangbuk-do, Gyeongsangnam-do, Jeju.

Among the 276 participants, 137 (49.8%) were male and 139 (50.4%) were female. The majority of the participants were 20–29 (33.0%), 30–39 (29.3%), and 40–49 (34.1%) years old. Participants aged 18–19 years were not excluded from the analysis because they were considered adults in South Korea. Regarding marital status, 134 participants (48.6%) were married and 142 participants (51.4%) were single. In terms of educational level, 201 participants (72.8%) had graduated from college or higher, followed by 47 college students (17%), and 28 high school graduates (10.1%). Professional/managerial/office jobs accounted for the largest number of occupations (141 participants, 51.1%), followed by homemakers and students (71 participants, 25.7%). A total of 142 participants (51.4%) had occupations that involved frequent interactions with others. A total of 134 participants (48.6%) had occupations involving relatively few interactions with others. Among them, 163 (59.1%) lived in the capital city. The capital area of South Korea is comprised of three districts: Seoul, Gyeonggi-do, and Incheon Metropolitan City. Non-capital areas refer to the remainder of the district.

### 3.2 Reliability and validity

We conducted factor analysis to evaluate the validity of the questionnaire. Principal component analysis and varimax rotation were performed. For validity verification, variables whose values were less than 0.6 or not bound by factors were excluded from the analysis. In addition, Cronbach’s alpha coefficient, which indicates the internal consistency between measurement items, was used for reliability analysis (Table 2).

**Table 2:** Reliability and validity.

Factors and items	Component						Cronbach’s alpha
	1	2	3	4	5	6	
IU01	**0.834**	0.122	0.117	0.120	0.078	0.125	0.819
IU02	**0.822**	0.164	0.118	0.197	0.165	0.140	
IU03	**0.663**	0.377	0.118	0.127	0.121	0.260	
Eff01	0.155	**0.855**	0.093	0.139	0.019	0.117	0.773
Eff02	0.231	**0.698**	0.191	0.206	0.166	0.165	
Eff03	0.176	**0.667**	0.306	−0.051	0.283	0.207	
IA01	0.097	0.300	**0.806**	0.144	0.170	0.149	0.798
IA02	0.190	0.160	**0.791**	0.146	0.270	0.151	
SDM01	0.091	0.173	0.011	**0.805**	0.207	0.189	0.697
SDM02	0.230	0.109	0.208	**0.658**	0.380	−0.101	
SDM03	0.303	0.024	0.366	**0.600**	−0.112	0.324	
Tra01	0.105	0.136	0.145	0.133	**0.827**	0.112	0.674
Tra02	0.164	0.156	0.255	0.240	**0.667**	0.205	
Usa01	0.197	0.242	0.132	0.024	0.286	**0.791**	0.757
Usa02	0.252	0.204	0.201	0.276	0.057	**0.756**	
Eigenvalue	2.257	2.147	1.778	1.768	1.672	1.634	
Variance (%)	15.050	14.311	11.852	11.789	11.147	10.893	
Cumulative percentage (%)	15.050	29.361	41.213	53.002	64.149	**75.041**	
# of Items	3/4	3/6	2/4	3/7	2/6	2/5	

IU, Intention to Use; Eff, Effectiveness; IA, Information Accuracy; SDM, Self-Determination; Tra, Transparency; Usa, Usability.

Six factors were derived from factor analysis: intention to use, effectiveness, information accuracy, self-determination, transparency, and usability. The maximum and minimum values were 0.855 and 0.600, respectively, and the analysis confirmed that all values were 0.6 or more (16, 19, 20). In addition, the eigenvalue, which constituted 75.041% of the total variance, was greater than 1.0. Cronbach’s alpha was used to verify reliability, which is the internal consistency of the variable. The maximum and minimum Cronbach’s alpha values were 0.819 and 0.674, respectively. Therefore, because all values were 0.6 or more (19, 21), it was considered reliable.

### 3.3 Overall user assessment of the KODARI app

**Figure 2:**
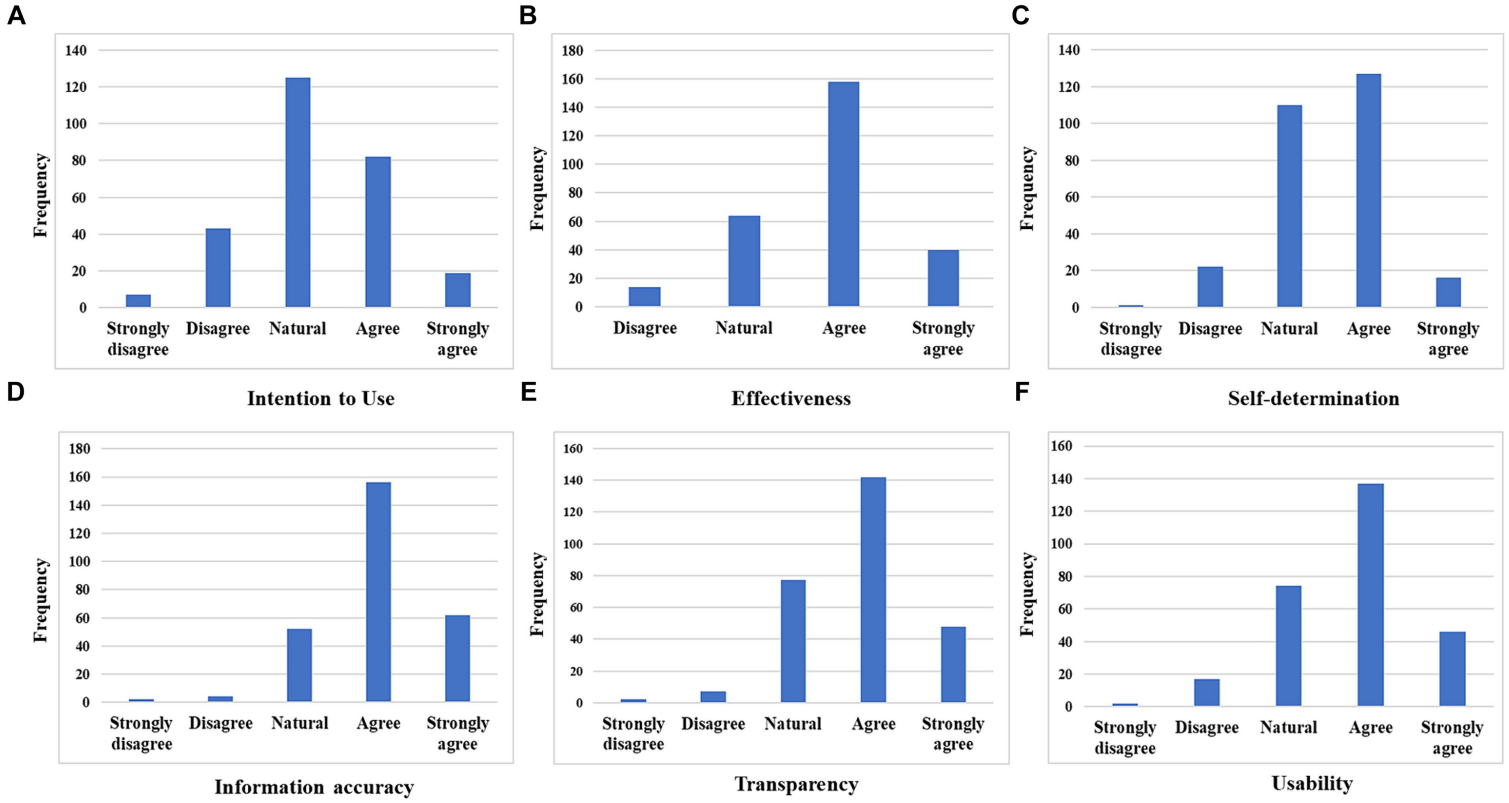
Overall assessment from the KODARI app users.

Figure 2 shows the overall evaluation of the KODARI application and assessment results for the six variables. The six variables were intention to use the KODARI app, effectiveness, self-determination, information accuracy, transparency, and usability. The six variables are tied based on the factor analyses presented in Table 2. The detailed assessment results from the KODARI app are included in Appendix Table A2.

First, when participants were asked whether they intended to use the app, 125 (45.3%) answered moderately. However, 101 participants (36.6%) responded that they were willing or extremely willing to use the KODARI app.

Effectiveness refers to the efficiency of an app in epidemiological investigations. Among the total respondents, 198 (71.7%) responded that the KODARI app would be useful for epidemiological investigations and would reduce the workload of epidemiological investigators.

Additionally, 143 participants (51.8%) responded positively to the app’s self-determination features, accounting for more than a majority of participants. A total of 110 (39.9%) and 253 (91.7%) participants expressed moderate and positive opinions, respectively.

Among the participants, 218 (79.0%) responded positively to the information accuracy question. The information accuracy variable had the least negative response (six participants, 2.8%) among the six variables.

Those who positively rated transparency, including 142 participants (51.4%) who responded “agree,” accounted for 68.8% (190 participants) of all participants. Finally, 183 participants responded positively to usability and 66.3% evaluated it positively.

### 3.4 Demographic differences among KODARI user assessments

The results of two independent sample *t*-tests were used to determine the differences between each variable (intention to use, effectiveness, self-determination, information accuracy, transparency, and usability) according to the participants’ demographic characteristics. First, the participants were divided into two groups based on their demographic characteristics: sex, marital status, occupational characteristics, and location (Table 1). Age was divided into two groups based on the age of 35 years, which was the mean age (34.88) of all participants. The average age of the respondents was 34.88 years, whereas their median age was 36 years: standard deviation (8.853), variance (78.372), skewness (−0.094), and kurtosis (−1.209) values. Age was skewed to the left, and the distribution of participants in their 20s and 40s had similar characteristics. Consequently, the sample was divided into subgroups of 35 years, which approximated the average age.

Educational level and occupation were excluded from the analysis because they could not be divided into two groups or based on the averages. Second, because the purpose of the KODARI app is to support epidemiological investigations, we used variables related to epidemiological investigations for the analysis: experience with epidemiological investigations and the form of epidemiological investigations (Table 1). Therefore, the demographic characteristics included seven variables: sex, age, marital status, occupational characteristics, location, experience with epidemiological investigation, and the form of epidemiological investigation. The summary details are presented in Table 3 and the full results are presented in Appendix Table A3.

**Table 3:** Two independent samples *t*-tests for demographic differences (summary version).

Variables			*N*	Mean	*SD*	*t*	Sig.
Sex	IA	Male	137	3.741	0.770	−2.075	**0.039** ^*^
		Female	139	**3.921**	0.668		
	IU	Male	137	**3.416**	0.791	2.559	**0.011** ^*^
		Female	139	3.161	0.865		
Age	IA	≤35	133	**4.030**	0.665	4.544	**0.000** ^***^
		>35	143	3.647	0.731		
	Tra	≤35	133	**3.707**	0.776	2.185	**0.030** ^*^
		>35	143	3.510	0.716		
Marital status	IA	Married	134	3.724	0.665	−2.418	**0.016** ^*^
		Single	142	**3.933**	0.765		
Occupational characteristics	SDM	Frequent	142	**3.617**	0.715	3.229	**0.001** ^**^
		Less	134	3.353	0.639		
Experience with epidemiological investigation	SDM	Yes	105	**3.603**	0.662	2.164	**0.031** ^*^
		No	171	3.419	0.700		

^*^p < 0.05, ^**^p < 0.01, ^***^p < 0.001. IU, Intention to Use, Eff: Effectiveness, IA: Information Accuracy, SDM: Self-Determination, Tra: Transparency, Usa: Usability.

The results showed a significant difference in information accuracy and intention to use, according to sex. For male (*M* = 3.741, SD = 0.770) and female (*M* = 3.921, SD = 0.668) participants, there was a significant difference in information accuracy (*t* = −2.075, *p* = 0.039) according to sex. In addition, for male (*M* = 3.416, SD = 0.791) and female (*M* = 3.161, SD = 0.865) participants, there was a significant difference in the intention to use (*t* = 2.559, *p* = 0.011). Females had higher information accuracy, whereas males had a higher intention to use (*p* < 0.05).

There were significant differences in information accuracy and transparency depending on the participants’ age. The mean and standard deviation of participants aged ≤35 years (*M* = 4.030, SD = 0.665) and > 35 years (*M* = 3.647, SD = 0.731) showed a significant difference in information accuracy (*t* = 4.544, *p* = 0.000) according to age, based on the age of 35 years. In addition, participants aged ≤35 years (*M* = 3.707, SD = 0.776) and those aged >35 years (*M* = 3.510, SD = 0.716) showed a significant difference in transparency (*t* = 2.185, *p* = 0.030). Participants under 35 years old had higher information accuracy and transparency (*p* < 0.05, *p* < 0.001).

Regarding to marital status, there was a significant difference in the information accuracy. The mean and standard deviation of married participants (*M* = 3.724, SD = 0.665) and single participants (*M* = 3.944, SD = 0.765) showed a significant difference in information accuracy (*t* = −2.418, *p* = 0.016) depending on marital status. Regarding marital status, single participants had a higher information accuracy (*p* < 0.05).

There were significant differences in self-determination according to occupational characteristics and experience in epidemiological investigations. Occupations involving frequent interactions with others (*M* = 3.617, SD = 0.715) and those involving relatively fewer interactions with others (*M* = 3.353, SD = 0.639) showed a significant difference in self-determination (*t* = 3.229, *p* = 0.001) according to occupational characteristics.

Finally, participants with experience in epidemiological investigations (*M* = 3.603, SD = 0.662) and those with no epidemiological investigation experience (*M* = 3.419, SD = 0.700) showed a significant difference in self-determination, depending on the presence or absence of epidemiological investigation experience (*t* = 2.164, *p* = 0.031). Therefore, participants with occupations involving frequent interactions with others and experience with epidemiological investigations had a higher self-determination (*p* < 0.01, *p* < 0.001).

### 3.5 Influencing factors on intention to use the KODARI app

Table 4 summarizes the results of this analysis. The model, which included all (five) independent variables, had an *F*-statistical value of 41.273 and a significance value of 0.000. Differences were considered statistically significant at significance level < 0.001. The coefficient of determination (*R*^2^) of this model was 0.433, and the independent variables of effectiveness, information accuracy, self-determination, transparency, and usability explained the change in the dependent variable of intention to use by 43.3%.

**Table 4:** ANOVA* for regression models (*n* = 276).

	Sum of squares	*df*	Mean square	*F*	Sig.
Regression	83.502	5	16.700	41.273	**0.000** ^***^
Residual	109.250	270	0.405		
Total	192.752	275			
*R*^2^(adj. *R*^2^) = 0.433(0.423)					

^***^p < 0.001. ^*^ANOVA: One-way Analysis of Variance.

Table 5 presents the results of the statistical significance analysis and contribution of each independent variable to the dependent variable. *Effectiveness* (*t* = 4.457, *p* = 0.000), *self-determination* (*t* = 4.504, *p* = 0.000), and *usabilit*y (*t* = 4.268, *p* = 0.000) had significant impacts on the dependent variable and intention to use the KODARI app. *Information accuracy* (*t* = 0.399, *p* = 0.690) and *transparency* (*t* = 0.488, *p* = 0.626) were rejected as a result of the analysis. According to the standardized coefficient, which represents the relative statistical contribution of the three independent variables, effectiveness (0.266), self-determination (0.258), and usability (0.252) affected the participants’ intention to use the KODARI app.

**Table 5:** Multiple regression analysis of intention to use the KODARI app (*n* = 276).

Independent variable	Unstandardized coefficients		Standardized coefficients	*t*-value	Sig.
	B	Std. Error			
(Constant)	−0.220	0.260			
Effectiveness	0.328	0.074	0.266	4.457	**0.000** ^***^
Self-determination	0.313	0.070	0.258	4.504	**0.000** ^***^
Information Accuracy	0.028	0.071	0.024	0.399	0.690
Transparency	0.032	0.065	0.028	0.488	0.626
Usability	0.264	0.062	0.252	4.268	**0.000** ^***^

^***^p < 0.001.

## 4 Discussion

We developed the KODARI app based on an *infectious disease self-management model*. In this study, we evaluated whether the KODARI app works properly in epidemiological investigations and in the prevention and overcoming of infectious diseases in the future, and evaluated factors that can lead to the continuous intention to use the app.

First, regarding the overall app evaluation, we found that participants were generally satisfied with the KODARI app. Regarding the intention to use, more participants said that they wanted to continuously manage and use health information related to infectious diseases as a preventive measure. Regarding effectiveness, more than half the participants said that the app would help with epidemiological investigations in the event of new infectious diseases in the future. The participants said that the app would have a positive effect on reducing the work of epidemiological investigators. These results are meaningful, as they prove that the epidemiological investigation support app is effective. More than half of the participants evaluated self-determination positively, that is, the app received positive evaluations that allowed users to store, retrieve, process, and delete data on their own. Studies have shown that individuals are reluctant to randomly store and use their information regarding COVID-19 (22, 23). This is an important function of the application that supports future epidemiological investigation. Information accuracy was the most positive variable evaluated by the participants. Participants stated that the information gathered or provided by the application was accurately recorded. Usability analyzes the ease and familiarity of an application’s interface or function. The findings show that usability received a positive evaluation from more than a majority of participants. This proves that the information provided by the KODARI application was accurate. This demonstrates that the app was easy to use. Information accuracy and usability are particularly important for infectious-disease–related applications, and these issues should be considered when developing related mHealth apps. Transparency considered whether the app required minimal personal data or access permissions and received a positive evaluation from the majority of participants. The KODARI application was developed to collect only a minimum amount of data, considering that individuals are generally passive in their sharing personal data.

Second, by analyzing whether there were differences in the six variables according to demographic characteristics, the findings showed that there were differences in information accuracy and intention to use according to sex. Females placed more importance on the accuracy of the information collected or provided by the app than males. Additionally, males were more willing to use the app and recommend it to acquaintances than females were. Participants aged under 35 years were more likely to perceive information accuracy and transparency to be higher. Additionally, the information accuracy was higher among single participants than among married participants. Thus, participants aged under 35 years and single appeared to place more emphasis on information accuracy than those aged over 35 years. This means that participants aged under 35 years may be more sensitive to digital technology or smartphone use than those aged over 35 years and may consider the accuracy of the information collected or provided by the app, such as location information, vaccination information, and symptom records, using global positioning system (GPS) technology more important than those aged over 35 years. It is possible that single participants have fewer family members than married participants, or that they have a wider range of activities. Therefore, single individuals may have more situations in which they rely on the information collected or provided by the app, and thus consider information accuracy as an important variable. Additionally, participants aged under 35 years were more likely to perceive that the application required minimal data or access than those aged over 35 years. This can be considered in relation to younger people’s sensitivity to personal-information protection (24). Minimum data usage and access were found to have a more significant effect on participants aged under 35 years, which future researchers should consider in the activation and diffusion of apps. The findings show that occupational characteristics and epidemiological investigation experiences significantly affected self-determination. Participants with occupations involving frequent interactions with others and those with experience in epidemiological investigations could determine and select data that could be saved and processed using the app. This means that participants with occupations involving frequent interactions with others may have a relatively greater fear of the confirmation of infectious diseases, such as COVID-19, than those without this interaction. Additionally, those who have undergone epidemiological investigations may be aware of the inconvenience in the epidemiological investigation process. During the initial COVID-19 epidemiological investigation, problems with unwanted personal information disclosure and the process of tracking contacts caused many inconveniences (25). Therefore, these two groups may place greater importance on directly storing, processing, retrieving, and sharing desired data through apps, which should be considered in future studies.

Third, effectiveness, self-determination, and usability were the main factors influencing the intention to use the KODARI. Participants were willing to use an app such as the KODARI app when considering infectious disease management or epidemiological investigation. Therefore, its effectiveness should be considered when developing an application for COVID-19 or other infectious diseases. Additionally, the intention to use the app was high when the data could be managed independently, including retrieval, storage, processing, and deletion. The usage rate of existing government- or provider-oriented apps is low because individuals are reluctant to use them (22, 26). App user data, including automatically collected personal information, put pressure on users. Self-determination should be considered essential when developing or supplementing an app, because this burden can negatively affect the intention to use an app. Therefore, to ensure the continued use of apps related to COVID-19 or other infectious diseases, self-determination must be considered when developing these apps. Finally, the findings showed that the easier the usability of the function or interface of the app, the higher the intention to use the app. Similar results have been reported in previous studies, which found that the function and interface of an application should be simple (27). Therefore, when developing or supplementing apps in the future, the interface and functionality of apps should be considered important factors.

### 4.1 Implications

This study has several important implications. First, in addition to the technology acceptance model (TAM) and unified theory of acceptance and use of technology model (UTAUT) (28–30), which are widely used in research on technology introduction or intention to use, we applied a new framework (15) for technologies related to COVID-19. Diverse technologies for infectious diseases such as COVID-19 have been introduced, but the situation is distinct. New attempts are required to evaluate technologies such as the KODARI app. Therefore, this study is meaningful in that it used a new evaluation tool that is different from existing studies, and confirmed the possibility of research with the variables used in this study. This study, which evaluated this app, is meaningful for developing the app and evaluating technology, considering the specificity of the infectious situation. However, further studies are required to evaluate technologies, such as the KODARI app. Therefore, this study contributes to the literature by using a new evaluation tool that is different from that used in previous studies, and confirms the possibility of further research with the variables used in this study.

Second, this study is meaningful because it directly developed an application related to infectious diseases and evaluated its effectiveness. Based on this, we found that an infectious disease management application for epidemiological investigations, such as KODARI and MEDARI apps, is helpful for epidemiological investigations and reduces the workload of epidemiological investigators. Scholars have conducted diverse studies on infectious diseases, such as COVID-19 (8, 31–35), and healthcare (36–39) apps and technologies. Yamamoto et al. reported that a personal health record (PHR)-based COVID-19 symptom-tracking app was an effective countermeasure against infectious diseases (40). However, few studies have evaluated the effectiveness of existing or directly developed infectious disease applications (41, 42). Therefore, this study is important because the researchers who participated in the app design process evaluated their effectiveness. Thus, the KODARI app provides a cornerstone for future application development.

Third, we found differences in sex, age, marital status, occupational characteristics, and experience with epidemiological investigations when evaluating the apps. This does not mean that apps should be designed differently based on specific occupations, experiences, or gender. It is desirable to use this to operate an app. For example, males had a higher intention to use the app than females. Based on these results, we will devise operational plans, such as promoting their application to female.

This study provides a basis for inventing or developing new applications in the event of future outbreaks of infectious diseases such as COVID-19 by identifying the factors affecting their introduction. The findings make an important contribution to literature on future infectious diseases.

### 4.2 Limitations

Despite this study’s meaningful results, it had some limitations. First, the total number of participants was 276, all of whom were KODARI app users; therefore, the results of this study have limitations in terms of generalization. We were unable to target all Koreans because of limited time and cost. As of 2023, the projected population of South Korea is approximately 51 million (43), and the cumulative number of confirmed COVID-19 cases is approximately 35 million (as of August 31, 2023; Korean Standard Time [KST]) (3). Future studies should focus on larger numbers of users. Second, there was a bias against vaccination. Among participants, 95.7% were confirmed to have completed the second- or higher-dose vaccination, and only 12 participants had never been vaccinated or only completed the first dose vaccination. The second-dose vaccination (fully vaccinated) rate for COVID-19 in South Korea is 86.6% (as of September 5, 2023, KST) (44). This study revealed a higher rate of secondary vaccination. The results would have been more meaningful if vaccination rates were similar. Third, the purpose of our modified questionnaire differs from that of the original evaluation framework (15). The original framework introduced a checklist that developers can use when creating apps. In this study, the questionnaire was modified for application to our study of app users and was administered to evaluate the effectiveness of the KODARI app, although we attempted to retain the intent of the existing framework as much as possible. Fourth, some domains and criteria were excluded when adapting the questionnaire and conducting factor analysis of the evaluation framework (15). In this study, this domain was referred to as a variable. There are seven domains in the evaluation framework (15): purpose, usability, information accuracy, organizational attributes, reputation (in this study, expressed as organizational attributes), transparency, privacy, user control, and self-determination (expressed as self-determination). Several criteria are presented for each domain. However, we used only four domains (usability, information accuracy, transparency, user control, and self-determination) and excluded three domains (purpose, organizational attributes, reputation, and privacy). Among the three excluded variables, privacy can affect an app’s effectiveness and intention to use. Privacy was excluded from this study because it was not included in the factor analysis. Therefore, further research should be conducted on purpose, organizational attributes, reputation, and privacy. Finally, we distributed the KODARI app on November 1, 2022. We targeted users who had used the app for more than a week (from November 2022 to the time of the survey). However, the results may differ depending on the period or amount of app use. In future research, it would be meaningful to target users who have been using the app for a long time.

## Data availability statement

The data analyzed in this study is subject to the following licenses/restrictions: Research data enquiries can be directed to the corresponding author. Requests to access these datasets should be directed to MR, rhomijung@dankook.ac.kr.

## Ethics statement

The studies involving humans were approved by the Institutional Review Board of Dankook University (IRB number: DKU 2023-02-011-004). The studies were conducted in accordance with the local legislation and institutional requirements. The ethics committee/institutional review board waived the requirement of written informed consent for participation from the participants or the participants’ legal guardians/next of kin because online informed consent to participate in this study.

## Author contributions

SS: Data curation, Writing – original draft, Formal analysis. JP: Conceptualization, Methodology, Validation, Writing – review & editing. MR: Conceptualization, Methodology, Writing – review & editing, Data curation¸ Funding acquisition, Investigation, Project administration, Supervision, Writing – original draft.
