# Supplementary material for: Effectiveness and intention to use a COVID-19 self-management app for epidemiological investigation: a web-based survey study
Source: Front Public Health. 2024 Mar 27;12:1343734. doi: 10.3389/fpubh.2024.1343734 (PMC11004299; doi:10.3389/fpubh.2024.1343734)
Supplement: Supplementary file 1 [file Data_Sheet_1.docx]

**Appendices**

**Appendix Table A1. Questionnaire items**

| **Usability** | 1. Is this app easy to use and has a friendly end-user interface?  2. Does the app send out a reasonable number of notifications?  3. Do you think the functions of this app are intuitive and easy to use?  4. Do you think this app is useful for users to manage infectious diseases on their own?  5. Do you think this app is useful for conducting infectious diseases epidemiological investigations? |
| --- | --- |
| **Information accuracy** | 1. Is the data (location information) generated by this app accurately recorded?  2. Is the information (symptom records, vaccination information, etc.) collected by the app accurately recorded?  3. Is the information (symptom records, vaccination information, etc.) provided by the app accurately recorded?  4. Does the app provide regular updates to contents based on improved research and recommendations? |
| **Transparency** | 1. Does the app inform the end user about the voluntary nature to participate?  2. Does the app highlight potential risks or side effects resulting from its use?  3. Does the app inform the end user what data is being collected?  4. Does the app require only minimal personal data of end-users?  5. Does the app require only minimal access permissions for using mobile phone functionalities (e.g. location, address book, camera)?  6. Is the app available for download from software download centers (Google Play, App store, etc.)? |
| **Self-determination** | 1. Do you think end-users act as the proprietors of the data generated from the app?  2. Does the app allow end-users to opt-in and decide which data can be stored or processed?  3. Does the app allow users to retrieve your data?  4. Can users get information about the results of the data analysis?  5. Does this app seek explicit consent from end-users before sharing data with third parties (such as epidemiological investigators)?  6. Does the app allow end-users to easily delete your data?  7. Does this app have the option to discontinue usage at any time (such as through withdrawal or account deletion)? |
| **Intention to use** | 1. Do you intend to use this app if there is a future outbreak of a new infectious disease that requires epidemiological investigation?  2. Would you like to continue using the KODARI app?  3. Do you want to recommend the KODARI app to others?  4. Do you intend to continuously store and manage your movement and health information related to infectious diseases for preventive purposes through the app? |
| **Effectiveness** | 1. What do you think this app can potentially contribute to mitigating infectious diseases like COVID-19?  2. Do you think this app would be helpful for epidemiological investigations during future outbreaks of new infectious diseases?  3. Compared to traditional epidemiological investigations, how much do you think this app would reduce the time required for conducting such investigations?  4. How much do you think using this app will reduce the stress caused by traditional epidemiological investigations?  5. Do you think that this app will reduce the workload of epidemiological investigations?  6. Would you like to reduce the workload of epidemiological investigators by using this app? |

**Appendix Table A2.** Overall assessment from KODARI app users.

| **Variables** | **Strongly disagree** | **Disagree** | **Neutral** | **Agree** | **Strongly agree** |
| --- | --- | --- | --- | --- | --- |
| Intention to use | 7 (2.5) | 43 (15.6) | 125 (45.3) | 82 (29.7) | 19 (6.9) |
| Effectiveness | 0 (0.0) | 14 (5.1) | 64 (23.2) | 158 (57.2) | 40 (14.5) |
| Self-determination | 1 (0.4) | 22 (8.0) | 110 (39.9) | 127 (46.0) | 16 (5.8) |
| Information accuracy | 2 (0.7) | 4 (1.4) | 52 (18.8) | 156 (56.5) | 62 (22.5) |
| Transparency | 2 (0.7) | 7 (2.5) | 77 (27.9) | 142 (51.4) | 48 (17.4) |
| Usability | 2 (0.7) | 17 (6.2) | 74 (26.8) | 137 (49.6) | 46 (16.7) |
| Total | 276 (100.0) | | | | |

**Appendix Table A3**. Two independent samples *t*-tests for demographic differences (full version).

| **Variables** | | | *N* | Mean | *SD* | *t* | Sig. |
| --- | --- | --- | --- | --- | --- | --- | --- |
| Sex | Usa | Male | 137 | 3.540 | 0.836 | -.144 | .886 |
|  |  | Female | 139 | 3.554 | 0.761 |  |  |
|  | IA | Male | 137 | 3.741 | 0.770 | -2.075 | **.039**** |
|  |  | Female | 139 | **3.921** | 0.668 |  |  |
|  | Tra | Male | 137 | 3.595 | 0.749 | -.223 | .823 |
|  |  | Female | 139 | 3.615 | 0.755 |  |  |
|  | SDM | Male | 137 | 3.465 | 0.746 | -.582 | .561 |
|  |  | Female | 139 | 3.513 | 0.634 |  |  |
|  | Eff | Male | 137 | 3.847 | 0.701 | .529 | .597 |
|  |  | Female | 139 | 3.803 | 0.660 |  |  |
|  | IU | Male | 137 | **3.416** | 0.791 | 2.559 | **.011**** |
|  |  | Female | 139 | 3.161 | 0.865 |  |  |
| Age | Usa | ≤35 | 133 | 3.613 | 0.889 | 1.310 | .191 |
|  |  | >35 | 143 | 3.486 | 0.699 |  |  |
|  | IA | ≤35 | 133 | **4.030** | 0.665 | 4.544 | **.000***** |
|  |  | >35 | 143 | 3.647 | 0.731 |  |  |
|  | Tra | ≤35 | 133 | **3.707** | 0.776 | 2.185 | **.030**** |
|  |  | >35 | 143 | 3.510 | 0.716 |  |  |
|  | SDM | ≤35 | 133 | 3.561 | 0.697 | 1.682 | .094 |
|  |  | >35 | 143 | 3.422 | 0.681 |  |  |
|  | Eff | ≤35 | 133 | 3.902 | 0.692 | 1.831 | .068 |
|  |  | >35 | 143 | 3.753 | 0.663 |  |  |
|  | IU | ≤35 | 133 | 3.281 | 0.843 | -.129 | .898 |
|  |  | >35 | 143 | 3.294 | 0.835 |  |  |
| Marital states | Usa | Married | 134 | 3.537 | 0.687 | -.198 | .843 |
|  |  | Single | 142 | 3.556 | 0.891 |  |  |
|  | IA | Married | 134 | 3.724 | 0.665 | -2.418 | **.016**** |
|  |  | Single | 142 | **3.933** | 0.765 |  |  |
|  | Tra | Married | 134 | 3.515 | 0.685 | -1.957 | .051 |
|  |  | Single | 142 | 3.690 | 0.801 |  |  |
|  | SDM | Married | 134 | 3.475 | 0.638 | -.327 | .744 |
|  |  | Single | 142 | 3.502 | 0.739 |  |  |
|  | Eff | Married | 134 | 3.789 | 0.619 | -.866 | .387 |
|  |  | Single | 142 | 3.859 | 0.733 |  |  |
|  | IU | Married | 134 | 3.376 | 0.743 | 1.716 | .087 |
|  |  | Single | 142 | 3.204 | 0.912 |  |  |
| Occupational characteristics | Usa | frequent | 142 | 3.588 | 0.868 | .877 | .381 |
|  |  | less | 134 | 3.504 | 0.716 |  |  |
|  | IA | frequent | 142 | 3.853 | 0.807 | .489 | .625 |
|  |  | less | 134 | 3.810 | 0.628 |  |  |
|  | Tra | frequent | 142 | 3.669 | 0.762 | 1.460 | .145 |
|  |  | less | 134 | 3.537 | 0.735 |  |  |
|  | SDM | frequent | 142 | **3.617** | 0.715 | 3.229 | **.001**** |
|  |  | less | 134 | 3.353 | 0.639 |  |  |
|  | Eff | frequent | 142 | 3.864 | 0.713 | .980 | .328 |
|  |  | less | 134 | 3.784 | 0.644 |  |  |
|  | IU | frequent | 142 | 3.380 | 0.857 | 1.906 | .058 |
|  |  | less | 134 | 3.189 | 0.808 |  |  |
| Location | Usa | Capital | 163 | 3.571 | 0.814 | .586 | .558 |
|  |  | Non-capital | 113 | 3.513 | 0.775 |  |  |
|  | IA | Capital | 163 | 3.862 | 0.729 | .838 | .403 |
|  |  | Non-capital | 113 | 3.788 | 0.719 |  |  |
|  | Tra | Capital | 163 | 3.647 | 0.780 | 1.121 | .263 |
|  |  | Non-capital | 113 | 3.544 | 0.706 |  |  |
|  | SDM | Capital | 163 | 3.462 | 0.719 | -.778 | .437 |
|  |  | Non-capital | 113 | 3.528 | 0.648 |  |  |
|  | Eff | Capital | 163 | 3.849 | 0.667 | .697 | .486 |
|  |  | Non-capital | 113 | 3.791 | 0.700 |  |  |
|  | IU | Capital | 163 | 3.321 | 0.844 | .801 | .424 |
|  |  | Non-capital | 113 | 3.239 | 0.828 |  |  |
| Experience with epidemiological investigation | Usa | Yes | 105 | 3.643 | 0.814 | 1.567 | .118 |
|  |  | No | 171 | 3.488 | 0.784 |  |  |
|  | IA | Yes | 105 | 3.914 | 0.722 | 1.490 | .137 |
|  |  | No | 171 | 3.781 | 0.724 |  |  |
|  | Tra | Yes | 105 | 3.662 | 0.745 | .986 | .325 |
|  |  | No | 171 | 3.570 | 0.754 |  |  |
|  | SDM | Yes | 105 | **3.603** | 0.662 | 2.164 | **.031**** |
|  |  | No | 171 | 3.419 | 0.700 |  |  |
|  | Eff | Yes | 105 | 3.873 | 0.685 | .921 | .358 |
|  |  | No | 171 | 3.795 | 0.678 |  |  |
|  | IU | Yes | 105 | 3.365 | 0.785 | 1.208 | .228 |
|  |  | No | 171 | 3.240 | 0.866 |  |  |
| Form of epidemiological investigation | Usa | Epidemiological | 30 | 3.817 | 0.771 | 1.391 | .167 |
|  |  | Simple | 75 | 3.573 | 0.825 |  |  |
|  | IA | Epidemiological | 30 | 3.817 | 0.688 | -.875 | .384 |
|  |  | Simple | 75 | 3.953 | 0.736 |  |  |
|  | Tra | Epidemiological | 30 | 3.617 | 0.751 | -.392 | .696 |
|  |  | Simple | 75 | 3.680 | 0.747 |  |  |
|  | SDM | Epidemiological | 30 | 3.789 | 0.669 | 1.839 | .069 |
|  |  | Simple | 75 | 3.529 | 0.649 |  |  |
|  | Eff | Epidemiological | 30 | 3.778 | 0.804 | -.813 | .420 |
|  |  | Simple | 75 | 3.911 | 0.633 |  |  |
|  | IU | Epidemiological | 30 | 3.566 | 0.739 | 1.584 | .116 |
|  |  | Simple | 75 | 3.289 | 0.795 |  |  |

**p* < .05, ***p* < .01, ****p* < .001

***IU***: Intention to Use, ***Eff***: Effectiveness, ***IA***: Information Accuracy, ***SDM***: Self-Determination, ***Tra***: Transparency, ***Usa***: Usability
